# Assessment of the Bacteria community structure across life stages of the Chinese Citrus Fly, *Bactrocera minax* (Diptera: Tephritidae)

**DOI:** 10.1186/s12866-019-1646-9

**Published:** 2019-12-24

**Authors:** Awawing Anjwengwo Andongma, Lun Wan, Yong-Cheng Dong, Yu-Lei Wang, Jin He, Chang-Ying Niu

**Affiliations:** 1 College of Plant Science & Technology, Huazhong Agricultural University, Wuhan, 430070 China; 2 State key laboratory of Agricultural Microbiology, Huazhong Agricultural University, Wuhan, 430070 China

**Keywords:** Fruit flies, *Bactrocera minax*, Symbiosis, Insect gut bacteria, 454 pyrosequencing

## Abstract

**Background:**

Symbiotic bacteria play a critical role in insect’s biology. They also offer great opportunities to improve on current pest management techniques. In order to exploit and integrate the roles played by the gut microbiota on pest management programs, a better understanding of the structural organization of the microbial community in the Chinese citrus fly *Bactrocera minax* is essential.

**Results:**

The results revealed a total of 162 OTUs at 97% similarity interval. The dominant bacteria phyla were Proteobacteria, Bacteroidetes, Antinobacteria and Firmicutes, with the Proteobacteria having the highest relative abundance (more than 80% in all life stages). There was also a shift in the dominant OTUs from the early developmental stages to the late developmental stages and adult stages in *B. minax*. These OTUs related to *Klebsiella pneumoniae*, *Providencia rettgeri* and *Enterobacter aerogenes*, respectively. Six bacteria OTU were shared by all the life stages. These belonged to the Enterobacteriaceae and the Enterococcaceae families.

**Conclusion:**

The common bacteria groups shared by all the life stages and other fruit flies could be important targets for further research. This should aim towards realizing how these bacteria affect the biology of the fly and how their relationship could be exploited in the development of sustainable management strategies against fruit flies*.*

## Introduction

The gut of most insects is colonized by microbial communities [1], which vary in diversity and function across the different insect groups [2]. Gut microbial colonization is affected by the morphology and physiology of the gut [1]. The origin of gut bacteria are from horizontal or vertical transmissions and some important bacteria groups have evolved to develop intimate relations with insects [3]. In social insects such as the honey bee, social interaction often leads to the transfer of important gut bacteria [3, 4]. In other insect groups such as the tephritids, the diversity and transmission patterns of important bacteria groups are poorly understood.

The gut of Tephritidae has been shown to be colonized by diverse symbiotic bacteria mainly belonging to the phylum Proteobacteria, Firmicutes and Bacteroidetes, Actinodetes and Tenericutes [5–10]. In tephritids, associations with symbiotic bacteria was first reported about a century ago [11], though the role these microorganisms play in their relationship with fruit flies was first reported about half a century later [12]. Over the years, many studies have focused on understanding the roles bacteria play in symbiotic relationships with tephritid flies. For example, *Wolbachia* was previously reported as an infective species manipulating the reproductive system of most insects [13, 14], but has recently be shown to have a mutualistic relationship with the bed bug [15]. Furthermore symbiotic bacteria has been shown to improve larval growth [12], provide nutritional supplements, which increased fecundity [16], affect longevity [17, 18], fix nitrogen [19] and improve sexual performance [20]. In the tephritid fly, symbiotic bacteria have been shown to improve sexual performance [21], detoxify insecticides [22] and could possibly be a lure for these flies [23]. Understanding the bacteria community structure in different fruit flies will be a first step towards realizing how the roles symbiotic bacteria plays could be exploited in developing an integrated pest management strategy for these flies.

The Chinese citrus fly *Bactrocera minax* (Enderlein) is considered unique in the Tephritidae family because of its long over-wintering pupal diapause and its single host, the citrus [24–27]. *B. minax* causes huge damage to citrus in China, Nepal, India and Bhutan where it is a major pest [26, 28]. Their overwintering capability with six months of long diapause makes it difficult to manage this pest [27, 29]. So far chemical control has shown to be ineffective probably due to the presence of Gluthinone S-tranferase which plays an important role in detoxification of several insecticides contributing to insecticide resistance in this pest [30]. In addition, commercially available pheromone attractive lures for *B. minax* males are unavailable [31]. Therefore, there is an urgent need to seek alternative control strategies for their management.

Given the huge potentials symbiotic bacteria offers for future pest management, this study was to conducted to understand the bacteria community structure associated with different life stages of *B. minax* using 454 FLX pyrosequencing. Bacteria were sampled from the egg, larva, pupa and adult of wild *B. minax*. We hypothesize that the different life stages will share some unique bacteria groups which are present in all life stages. These unique groups could be suitable target for further research in pest management programs.

## Methods

### Sample collection

Insect samples were collected from citrus orchards in Yichang, Hubei Province of China (30 °4́3́ N 111 ° 17′ E). Preliminary studies from our lab showed that *B. minax* was the only fruit fly specie present in this location. First instar (BM1L), second instar (BM2L), third instar (BM3L) larvae and eggs (BME) were collected from the infested fruits from July to November, 2012. Pupae (BMP) were dug from the soil in December 2012, they were  easily recognized from their extra-large size compared to that of *B. dorsalis*. The adults were collected using traps and protein baits in March 2013. Live male (BMM) and female (BMF) flies were allowed to starve for at least 12 h before gut dissection to clear the gut of allochthonous species. Ethical clearance was not required before insect collection as *B. minax* is indigenous to China.

### Insect dissection and DNA extraction

Prior to gut dissection, the adult and larva were anesthetized by chilling at − 20 °C for 10–20 min. Total DNA was extracted from a batch of 50 insects per life stage. Each life stage (BME, BM1L, BM2L, BM3L, BMP, BMM, and BMF) were sterilized in 70% ethanol for 2 to 5 min, rinsed thrice in sterile distilled water before dissection to remove the whole gut. The samples that were used for DNA extraction included the whole gut of the adult, second and third instar larva (excluding the malpighian tubules), the whole egg, first instar larva and pupae (without the puparium). Dissection was carried out under sterile conditions in a laminar flow hood using a stereomicroscope. The different samples were dissected in sterile distilled water on a sterilized glass plate using a pair of sterile tweezers. After sterilizing the pupal case, the puparium was carefully remove with a pair of sterile tweezer. Total genomic DNA was extracted using the CTAB protocol as previously reported by [5].

### PCR amplification, amplicon quantification, pooling and pyrosequencing

Barcoded broadly conserved primers 27F_5’ CCTATCCCCTGTGTGCCTTGGCAGTCTCAGAGAGTTTGATCCTGGCTCAG-3′, and 533R_5′-CCATCTCATCCCTGCGTGTCTCCGACGACTNNNNNNNNTTACCGCGGCTGCT GGCAC − 3′, were used for PCR amplification of ~ 536 bp of variable region V1–3 of the 16S rRNA gene. These primers were modified to contain the A and B sequencing adaptors (454 Life Sciences) underlined in the above primer sequences. Eight base pair specific barcodes are represented by the Ns in the above sequence.

PCR reactions were carried out in 15 μL reactions in triplicate, and each reaction tube contained 0.2 mM of the forward and reverse primer, about 5 ng of template DNA, 1 X PCR reaction buffer, 1 U of *Pfu* DNA polymerase (MBI. Fermentas, USA). PCR was carried out under the following conditions: an initial denaturation step at 94 °C for 1 min, then 25 cycles of 94 °C for 30 s, 55 °C for 30 s and 72 °C for 1 min and a final extension phase of 72 °C for 10 min. The PCR products were subjected to electrophoresis on a 1.2% (w/v) agarose gel and stained with ethidium bromide to determine the presence and yield of the fragment of interest. Later, the PCR products were purified with a DNA gel extraction kit (Axygen, China). PCR product concentration was checked using a Quant-iT PicoGreen double-stranded DNA assay (Invitrogen, Germany) and quality control was carried out using an Agilent 2100 Bioanalyzer (Agilent, USA). Equimolar amount of DNA samples from the three replicates from each life stage were pooled and used for amplicon PCR. This was done in order to maximize the diversity of bacteria from the population sample rather than from an individual sample [5, 7]. Amplicon pyrosequencing was carried out from the A-end using a 454 Roche sequencing primer kit on a Roche Genome Sequencer GS FLX Titanium platform at National Human Genome Center at Shanghai, China.

### Data analysis

Community analysis was performed with Mothur [32] using the standard pipeline described at www.mothur.org/wiki/454_SOP, accessed April 1st 2015 as follows; the quality reads were extracted from the SFF file and the sequences were grouped according to barcode and primer. Sff files were trimmed based on sequence quality using the shhh.flows script and sequences were trimmed to eliminate those with more than two primer mismatches, any mismatch in the barcode, more than eight bases homopolymers or less than 200 bp. Unique sequences were aligned using the SILVA [33] reference alignment and sequences within 1–2 bp of a more abundant sequence were preclustered together. Chimeric reads were identified and removed using Uchime. Unique sequences were clustered into 162 OTU based of 3% difference. Operational taxonomic units (OTUs) were classified using the RDP training set version 9 [34] database. A BLAST was further carried out on NCBI nucleotide collection (nr/nt) using the megablast algorithm to obtain more information on taxonomic identity of the 7 most abundant OTU. Core bacterial OTU shared by the different life stages were identified by comparing OTUs from the different life stages. The heat-map of beta diversity indices was constructed using the Perl and SVG software. Bar charts showing percentage abundance was constructed on Origin 9.0 (Electronic Arts Inc., Rrdwood, California, USA) software. Alpha Diversity Indices including ACE, Chao and Shannon were calculated using QIIME [35] “alpha_diversity.py” script (http://qiime.org/scripts/alpha_diversity.html). Rarefaction curves were also constructed using the QIIME “alpha_rarefaction.py” script (http://qiime.org/scripts/alpha_rarefaction.html. Principle coordinate analysis was constructed on R [36].

## Results

### 16S rRNA bar-coded amplicon pyrosequencing and clustering into operational taxonomic units (OTU)

A total of 57,888 sequences were obtained from the seven *B. minax* samples (BM1L, BM2L, BM3L, BME, BMP, BMM, BMF) sequenced. After quality filtering and removal of chimeric sequences, a total of 54,581 sequences remained, this corresponded to 621 unique sequences. The estimated number of OTUs at 97% obtained from all the *B. minax* life stages was 162 (Additional file 1).

### Bacterial diversity and abundance

**Fig 1:**
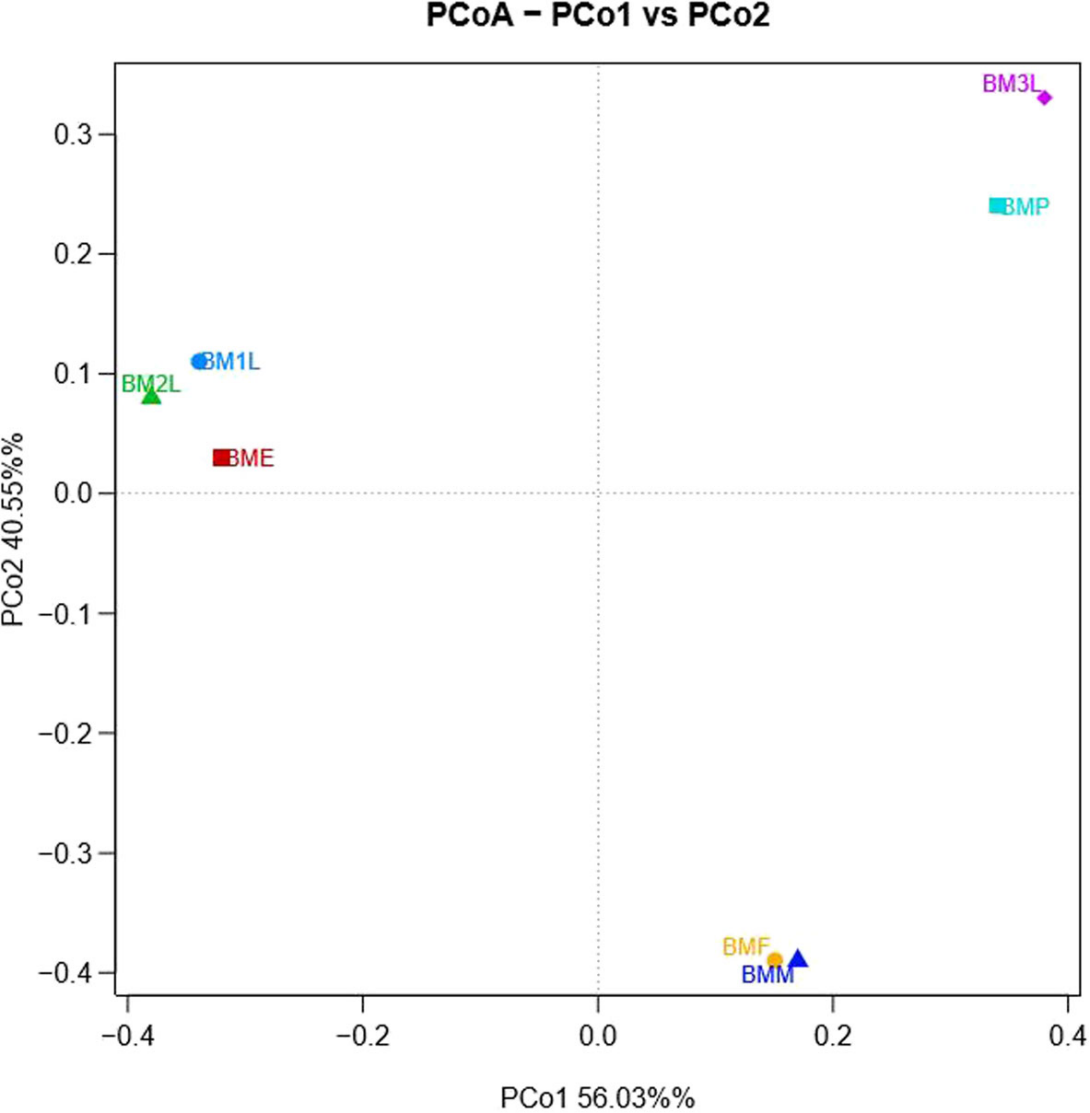
Comparison of bacterial communities associated with different developmental stages of *Bactrocera minax*. Principal Coordinate Analysis (PCoA) was generated with OTUs (at 3% dissimilarity) present in the different clone libraries; BME- *B. minax* egg, BM1L- *B. minax* first instar larva, BM2L- *B. minax* second instar larva, BM3L- *B. minax* third instar larva, BMP- *B. minax* pupa, BMF- *B. minax* adult female, BDM- *B. minax* adult male. Additional file 2 gives the PCoA loading values

The Chao and ACE richness indices show that amongst the *B. minax* samples, the egg stage (BME) appears to have the richest gut bacterial community. The Shannon and Simpson indices also revealed more diversity in early life stages (BME and BM1L) when compared with the later life stages (Table 1). The Chao1 and ACE did not equal the observed number of OTU in both samples suggesting that sampling in both did not reach saturation. The rarefaction curve did not asymptote. These results indicate the presence of some rare and unidentified groups in both samples which might have not been detected as a results of the limitations of the PCR technique used in this study [37]; however, coverage estimates appear to be high for all the samples (Table 1). Principal coordinate analysis revealed variations across the life cycle of the *B. minax*. Generally, the extent of variation correlated with the habitat as the egg, first instar and second instar larva were closely related and different from all other groups. On the other hand, the adult life stages were also closely related. An exception to this was the third instar larva, which was more related to the pupal stage, and these two were distantly related to all other groups (Fig. 1). The loading values across the different life stages of *B. minax*, that contribute to the pattern observed in the PCoA in Fig. 1 are shown in Additional file 2.

**Table 1:** Richness and diversity estimation of the 16S rRNA gene libraries from the pyrosequencing analysis of bacteria associated with life-stages of the Chinese citrus fly *Bactrocera minax*

Sample	Cutoff	Observed OTUs	Ace	Chao1	Shannon	Simpson	Coverage
BME	0.3	58	68.12	65.33	2.46	0.64	1.00
BM1L	0.3	56	62.97	62.88	2.43	0.66	1.00
BM2L	0.3	25	29.07	26.25	1.16	0.42	1.00
BM3L	0.3	17	22.21	20.00	0.53	0.17	1.00
BMP	0.3	44	55.52	53.43	1.84	0.61	1.00
BMF	0.3	35	50.63	48.20	2.35	0.75	1.00
BMM	0.3	35	40.54	37.10	2.28	0.74	1.00

### Taxonomic composition of bacteria in the Chinese citrus fly

**Fig 2:**
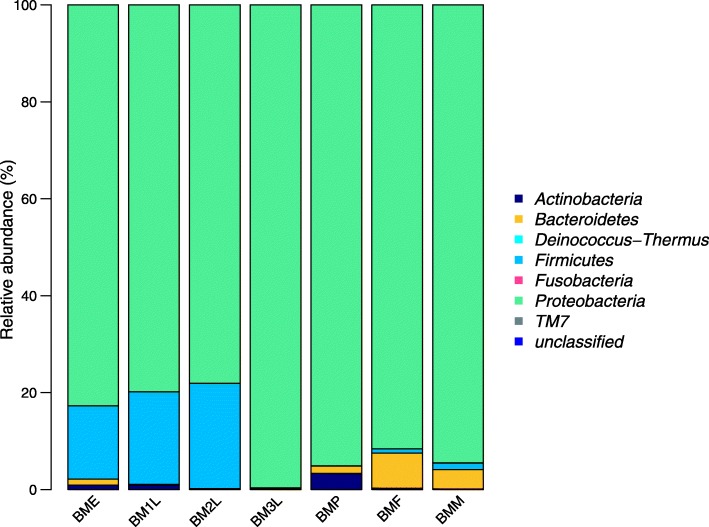
Relative bacteria composition of the different phyla in the guts of different developmental stages of *Bactrocera minax***:** BME- *B. minax* egg, BM1L- *B. minax* first instar larva, BM2L- *B. minax* second instar BM3L- *B. minax* third instar larva, BMP- *B. minax* pupa, BMF- *B. minax* female, BMM- *B. minax* male

The 162 OTU realized from the different seven *B. minax* samples could be grouped into six different bacteria phyla and some unclassified groups. These phyla include; Proteobacteria, Firmicutes, Bacteroidetes, Antinobacteria, Fusobacteria, and TM7 (Fig. 2). Amongst these, the Proteobacteria was the most abundant in all life stages having approximately 80% reads in all life stages. Firmicutes was also dominant in eggs, first and second instar larvae having an abundance of at least 15% in these stages and less than 2% in the other life stages. Actinobacteria comprised 3% of the sequences in the pupae and less than 1% in all other life stages. Bacteroidetes were relatively abundant in the adult female (7% of sequences) and were completely absent in third instar larvae.

**Fig 3:**
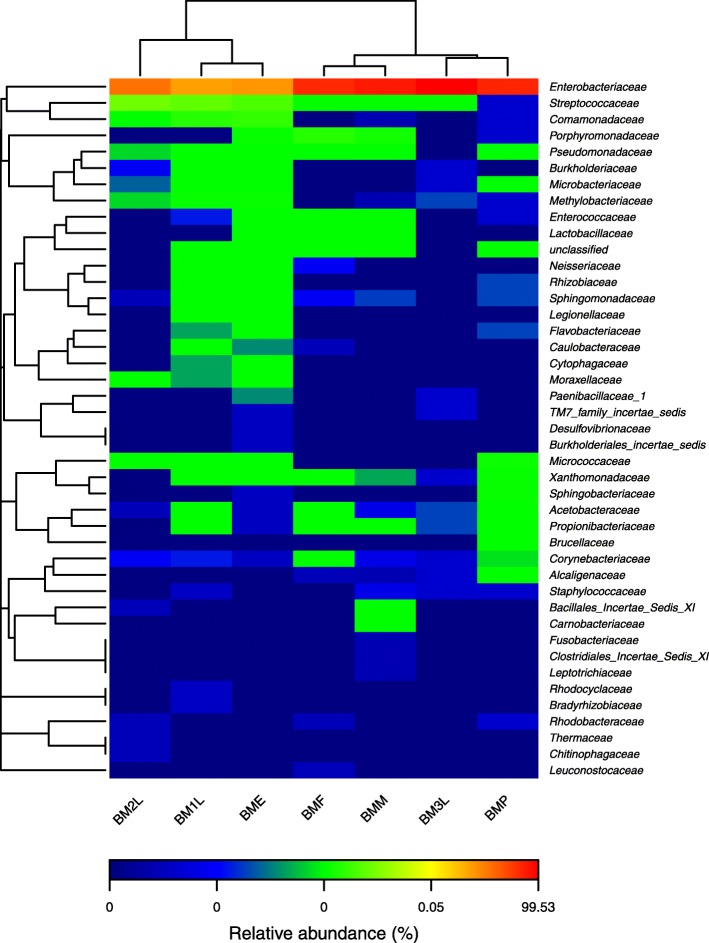
Heat map showing bacterial family frequency distribution across the seven different life stages. The heat map plot depicts the relative percentage of each bacterial family (variables clustering on the Y-axis) within each sample (X-axis clustering). The relative values for bacterial family are depicted by color intensity with the legend indicated at the bottom of the figure. Clusters based on the distance of the seven samples along the X-axis and the bacterial families along the Y-axis are indicated in the upper and left of the figure, respectively

The most abundant OTUs was represented by an Enterobacteriaceae (Fig. 3, Additional file 3) with a BLAST search showing *Klebsiella pneumoniae* strain PBCUK21 (accession number LC216325.1) (Table 2) as its closest match. It comprised at least 55% of the bacteria present in the egg, first and second instar larva. However, this population decreases to less than 1% in the pupa before increasing to about 21% in the adults. The second most abundant OTU also belonged to the Enterobacteriaceae family. A Blastn search revealed *Providencia rettgeri* strain RB151 (accession number: CPO17671.1) as the closest match. This OTU bore the most abundant reads in the third instar larvae (90%) and pupae (47%). Although it was present in all the other life stages, it only represented less than 1% of the total OTU. Similarly, the third most abundant OTU also belonged to the Enterobacteriaceae family. A Blastn search revealed its closest match to be *Enterobacter aerogenes* strain X-2 (accession number: 508303.1). This OTU was dominant in the adult life stage (about 37%) and almost 0 % in all other life stages. Though the most abundant OTUs belonged to the Enterobacteriaceae family, species abundance varied across the life cycle of *B. minax*. The eggs, first and second instar larvae were dominated by *K. pneumoniae* and *Lactococcus lactis*, the larvae and pupae by *Providencia rettgeri* and the adults by *Enterobacter aerogenes* and *Citrobacter freundii* (Table 2).

**Table 2:** Abundance of 16S rRNA gene amplicons across developmental stages of *B. minax*, expressed as % of total in each life stage

OTU	Best match gene #	Best match gene %	Best match gene ID	BME	BM1L	BM2L	BM3L	BMP	BMF	BMM
Otu001	LC216325.1	100	*Klebsiella pneumoniae*	59.36	56.03	72.92	8.76	0.85	21.37	23.38
Otu002	CP017671.1	100	*Providencia rettgeri*	0.23	0.62	0.39	90.53	47.67	0.23	0.38
Otu003	KY508303.1	100	*Enterobacter aerogenes*	3.72	0.47	0.24	0.03	0.22	36.53	37.19
Otu004	KU992685.1	100	*Citrobacter freundii*	2.87	0.33	0.01	0.02	0.21	25.31	25.56
Otu005	LC214977.1	100	*Lactococcus latis*	14.74	19.03	21.67	0.22	0.02	0.35	0.06
Otu006	KU942503.1	100	*Morganella morganii*	0.00	0.05	0.00	0.09	40.76	0.05	0.13
Otu007	KX259561.1	100	*Serratia marcescens*	2.47	8.75	3.60	0.02	0.03	0.01	0.03
Others				16.61	14.72	1.17	0.33	10.24	16.15	13.27

### Core intestinal microbiota

The bacteria present in the BM libraries were investigated for the presence of core gut microbiota shared by all the life stages. A total of six bacterial OTUs (1, 2, 3, 4, 5 and 7) were detected in all samples (Table 2). Five of these belong to the Enterobacteriaceae family (Proteobacteria) and one belonged to the Enterococaceae family (Table 2).

## Discussion

In this study the microbiome associated with *B. minax* was identified and characterized by 454 FLX pyrosequencing. This study reports for the very first time the bacterial diversity and abundance associated with the immature stages of *B. minax* using a non-culture-based approach. A previous study on the bacterial community of this fruit fly focused only on the adult flies [38]. The results from this study suggest that Enterobacteriaceae dominates all life stages of the fly. This family has also been reported to be dominant in other tephritids [9, 10, 23, 39, 40]. In addition *Klebsiella*, *Citrobacter*, *Enterobacter* and *Serratia* have been reported as the dominant genera found in the gut and reproductive system of adult *B. minax* [38]. The present study also reports similar findings.

In this study, the majority of the sequence reads belong to Proteobacteria, Firmicutes, Bacteroidetes and Actinobacteria. *B. minax* is oligophagous with citrus being its only host plant. Different bacterial species previously described to be associated with citrus plants belong to Proteobacteria, Firmicutes, Bacteroidetes and Actinobacteria with the Proteobacteria being the most abundant [41, 42]. The host plant is known to have significant effects on insect gut bacteria structure [43]. Symbiotic bacteria of fruit flies have also been reported to be transmitted horizontally and vertically [44, 45]. This suggests that the origin of some of the bacteria found in the gut could be from environmental samples (plant host). Bacteria species which have established unique transmission patterns in fruit flies are those which have co-evolved with the flies. If these bacteria groups could be identified in further studies, they will constitutes important target which could be exploited in pest management programs. It is very likely that these bacteria groups could be among the dominant species or core bacteria groups shared by the different life stages.

Host diet and phylogeny shape mammal associated gut resident bacteria [46, 47]. Diet also affects the gut bacteria community structure of some insects including *Drosophila* [43], gypsy moth [48] and cotton bollworm [49]. The bacterial community of the egg clustered with that of the first and second instar larva while that of the third instar larva clustered with the pupal. The adult male and female clustered together. This suggests that the larvae, pupae and adults have different resident bacteria within the Enterobacteriaceae, which although are different they have a close taxonomic relationship. This has also been reported in *Ceratitis capitata* [10]. The results from our study suggest that the bacterial community structure of the *B. minax* is not only shaped by the host’s diet but also other factors [50]. The observed variation in bacteria community across life stages reported in this study is not uncommon in tephritids as these variations have also been reported in the *B. dorsalis* [5] and the *C. capitata* [10]. However, for an oligophagous pest such as the *B. minax* that feeds solely on citrus. It is surprising that the egg and first instar larva recorded the highest bacteria diversity. Re-localization of some bacteria species to different organs as the insect matures may be a possible reason for the observed differences. A previous study had reported that, *B. minax* host a higher diversity of gut bacteria in their ovaries and testes when compared to the gut [38].

Different members of these Enterobacteriaceae group have been shown to play different fitness roles in fruit flies. In *B. oleae* for example symbiotic bacteria *Candidatus Erwinia dacicola* helps the young larvae to overcome host defence [51]. The most dominant OTUs in the egg, first and second instar larva were related to *Klebsiella pneumoniae* LC216325.1. *Klebsiella spp* has been isolated from the gut of other tephritids [40, 52]. Bacteria belonging to this genus could act as an attractive lure for fruit flies [53, 54] and improve on mating competiveness in fruit flies [55]. It has also been shown to have nitrogen fixing properties [19].

The dominant OTU in the pupae is related to *P. rettgeri* (Table 2). Similar member belonging to this genus has been identified in different tephritids [40, 52, 56]. *B. minax* undergoes diapause during harsh winter [27, 29]. The high relative abundance of *Providencia* in third instar larvae and pupae, but not other life stages, suggests that it might be playing a role in diapause. A shift in gut bacteria community structure has been reported in diapausing insects [57] and obese humans [58] who have large fat reserves. *Providencia* has been shown to have nitrifying-denitrifying functions [59], thereby converting ammonia into less toxic waste. Speculatively *Providencia* present in the BM3L and BMP are likely to help the insect remove metabolic waste during the long diapause period. However, more research needs to be carried out to confirm this hypothesis.

From our results the OTUs 1, 2, 3, 4, 5 and 7 were shared by all life stages of the fly (Table 2). This suggests the possibility of vertical transmission of these bacteria. Vertically transmitted bacteria in tephritids have been reported to be present in the larval stage and maintained throughout the adult stage [45]. In addition, 4 of these core OTUs found in *B. minax*, were also present in the oriental fruit fly [5], suggesting that they may play key roles in the biology of the fly.

## Conclusion

This study reports for the very first time the symbiotic bacteria present in the gut of the different developmental stages of the Chinese citrus fly and compares this population with the microbiome present in the adult life stages. The results revealed that the gut of the Chinese citrus fly harbors a large diversity of microorganisms belonging to 8 different phyla. Though Proteobacteria phyla dominated in all life stages, there was a shift in the most abundant OTUs from early development to maturity. Core bacteria groups shared by all life stages and different fruit flies belonged to the Enterobacteriaceeae and the Enterococcaceae families. These species have also been found in other fruit fly groups and some have been reported to play significant roles in the biology of other fruit flies. These could be important targets for the further research on how symbiotic bacteria could be exploited in the management of fruit flies.

## Supplementary information

**Additional file 1. **Relative abundance of taxa in the 16S rRNA libraries from different developmental stages of *Bactrocera minax***:** BME- *B. minax* egg, BM1L- *B. minax* first instar larva, BM2L- *B. minax* second instar BM3L- *B. minax* third instar larva, BMP- *B. minax* pupa, BMF- *B. minax* female, BMM- *B. minax* male. Classification results were obtained from sequence alignment against RDP training set version 9 [34] and can be displayed for different taxonomic levels (Phylum; Class; Order; Family; Genus; Operational taxonomic units created at 97% sequence similarity)

**Additional file 2. **Loading values across the different life stages of *B. minax*, that contribute to the pattern observed in the PCoA in Fig. 1. Loading values were calculated by weighted Unifrac Principal Coordinate Analysis (PcoA).

**Additional file 3. **Relative bacteria composition of the different families in the guts of different developmental stages of *Bactrocera minax***:** BME- *B. minax* egg, BM1L- *B. minax* first instar larva, BM2L- *B. minax* second instar BM3L- *B. minax* third instar larva, BMP- *B. minax* pupa, BMF- *B. minax* female, BMM- *B. minax* male

## Data Availability

Pyrosequencing data has been submitted to the GenBank (SRA) database as a file under the accession number SRP126595.

## Abbreviations

BDM: *Bactrocera minax* adult male
BM1L: *Bactrocera minax* first instar larva
BM2L: *Bactrocera minax* second instar larva
BM3L: *Bactrocera minax* third instar larva
BME: *Bactrocera minax* egg
BMF: *Bactrocera minax* adult female
BMP: *Bactrocera minax* pupa
CTAB: Cetyl trimethylammonium bromide
DGGE: Denaturing Gradient Gel Electrophoresis
DNA: Deoxyribonucleic acid
NCBI: National Center for Biotechnology Information
NGS: Next generation sequencing
OTU: Operational Taxonomic Unit
PBS: Phosphate Buffered Saline
PCoA: Principal Coordinate Analysis
PCR: Polymerase Chain reaction
RDP: Ribosomal Database Project
RDP: Ribosomal database Project
rRNA: Ribosomal ribonucleic acid

## References

[1] Dillon R, Dillon V (2004). The gut bacteria of insects: nonpathogenic interactions. Annu Rev Entomol.

[2] Douglas AE (2015). Multiorganismal insects: diversity and function of resident microorganisms. Annu Rev Entomol.

[3] Engel P, Moran NA (2013). The gut microbiota of insects–diversity in structure and function. FEMS Microbiol Rev.

[4] Powell J. Elijah, Martinson Vincent G., Urban-Mead Katherine, Moran Nancy A. (2014). Routes of Acquisition of the Gut Microbiota of the Honey Bee Apis mellifera. Applied and Environmental Microbiology.

[5] Andongma AA, Wan L, Dong Y-C, Desneux N, White JA, Niu C-Y. Pyrosequencing reveals a shift in symbiotic bacteria populations across life stages of *Bactrocera dorsalis*. Sci Rep. 2015;5. 10.1038/srep09470 PMC5380164 25822599

[6] Wang A, Yao Z, Zheng W, Zhang H. Bacterial communities in the gut and reproductive organs of *Bactrocera minax* (Diptera: Tephritidae) based on 454 pyrosequencing. 2014:9(9):e106988. 10.1371/journal.pone.0106988 PMC4162550 25215866

[7] Morrow J, Frommer M, Shearman D, Riegler M. The microbiome of field-caught and laboratory-adapted Australian tephritid fruit fly species with different host plant use and specialisation. Microb Ecol. 2015:1–11. 10.1007/s00248-015-0571-1 25666536

[8] Behar A, Yuval B, Jurkevitch E (2008). Community structure of the mediterranean fruit fly microbiota: seasonal and spatial sources of variation. Isr J Ecol Evol.

[9] Capuzzo C, Firrao G, Mazzon L, Squartini A (2005). Girolami V: ‘*Candidatus Erwinia dacicola*’, a co-evolved symbiotic bacterium of the olive fly *Bactrocera oleae* (Gmelin). Int J Syst Evol Microbiol.

[10] Aharon Yael, Pasternak Zohar, Ben Yosef Michael, Behar Adi, Lauzon Carol, Yuval Boaz, Jurkevitch Edouard (2012). Phylogenetic, Metabolic, and Taxonomic Diversities Shape Mediterranean Fruit Fly Microbiotas during Ontogeny. Applied and Environmental Microbiology.

[11] Petri L (1910). Untersuchung uber die darmbakterien der olivenfliege. Zentbl Bakteriol.

[12] Hagen KS (1966). Dependence of the olive fly, *Dacus oleae,* larvae on symbiosis with *Pseudomonas savastanoi* for the utilization of olive. Nat.

[13] Hilgenboecker K, Hammerstein P, Schlattmann P, Telschow A, Werren JH (2008). How many species are infected with Wolbachia?–a statistical analysis of current data. FEMS Microbiol Lett.

[14] Werren JH, Baldo L, Clark ME (2008). Wolbachia: master manipulators of invertebrate biology. Nat Rev Microbiol.

[15] Nikoh N, Hosokawa T, Moriyama M, Oshima K, Hattori M, Fukatsu T (2014). Evolutionary origin of insect–Wolbachia nutritional mutualism. Proc Natl Acad Sci.

[16] Ben-Yosef Michael, Aharon Yael, Jurkevitch Edouard, Yuval Boaz (2010). Give us the tools and we will do the job: symbiotic bacteria affect olive fly fitness in a diet-dependent fashion. Proceedings of the Royal Society B: Biological Sciences.

[17] Ben-Yosef M, Behar A, Jurkevitch E, Yuval B (2008). Bacteria-diet interactions affect longevity in the medfly *Ceratitis capitata*. J Appl Entomol.

[18] Behar A, Yuval B, Jurkevitch E (2008). Gut bacterial communities in the Mediterranean fruit fly (*Ceratitis capitata*) and their impact on host longevity. J Insect Physiol.

[19] Behar A, Yuval B, Jurkevitch E (2005). Enterobacteria-mediated nitrogen fixation in natural populations of the fruit fly *Ceratitis capitata*. Mol Ecol.

[20] Ami EB, Yuval B, Jurkevitch E (2010). Manipulation of the microbiota of mass-reared Mediterranean fruit flies *Ceratitis capitata* (Diptera: Tephritidae) improves sterile male sexual performance. Int Soc Microb Ecol J.

[21] Gavriel S, Jurkevitch E, Gazit Y, Yuval B (2011). Bacterially enriched diet improves sexual performance of sterile male Mediterranean fruit flies. J Appl Entomol.

[22] Cheng D, Guo Z, Riegler M, Xi Z, Liang G, Xu Y (2017). Gut symbiont enhances insecticide resistance in a significant pest, the oriental fruit fly *Bactrocera dorsalis* (Hendel). Microbiome.

[23] Wang H, Jin L, Peng T, Zhang H, Chen Q, Hua Y (2014). Identification of cultivable bacteria in the intestinal tract of *Bactrocera dorsalis* from three different populations and determination of their attractive potential. Pest Manag Sci.

[24] Allwood AJ, Chinajariyawong A, Drew R, Hamacek E, Hancock D, Hengsawad C, Jipanin J, Jirasurat M, Krong CK, Kritsaneepaiboon S. Host plant records for fruit flies (Diptera: Tephritidae) in South East Asia: Department of Biological Sciences, National University of Singapore; 1999. accessed Aug 2016.

[25] Dorji C, Clarke AR, Drew RAI, Fletcher BS, Loday P, Mahat K, Raghu S, Romig MC (2006). Seasonal phenology of *Bactrocera minax* (Diptera: Tephritidae) in western Bhutan. Bull Entomol Res.

[26] Zhou X-W, Niu C-Y, Han P, Desneux N (2012). Field evaluation of attractive lures for the fruit fly *Bactrocera minax* (Diptera: Tephritidae) and their potential use in spot sprays in Hubei Province (China). J Econ Entomol.

[27] Dong Y-C, Wang Z-J, Clarke AR, Pereira R, Desneux N, Niu C-Y (2013). Pupal diapause development and termination is driven by low temperature chilling in *Bactrocera minax*. J Pest Sci.

[28] Wang X, Luo L (1995). Research progress in the Chinese citrus fruit fly. Entomol Knowl.

[29] Dong Yongcheng, Desneux Nicolas, Lei Chaoliang, Niu Changying (2014). Transcriptome Characterization Analysis of Bactrocera minax and New Insights into Its Pupal Diapause Development with Gene Expression Analysis. International Journal of Biological Sciences.

[30] Chen E-H, Dou W, Hu F, Tang S, Zhao Z-M, Wang J-J (2012). Purification and biochemical characterization of glutathione s-transferases in *Bactrocera minax* (Diptera: tephritidae). Fla Entomol.

[31] Drew RA, Dorji C, Romig MC, Loday P (2006). Attractiveness of various combinations of colors and shapes to females and males of *Bactrocera minax* (Diptera: Tephritidae) in a commercial mandarin grove in Bhutan. J Econ Entomol.

[32] Schloss PD, Westcott SL, Ryabin T, Hall JR, Hartmann M, Hollister EB, Lesniewski RA, Oakley BB, Parks DH, Robinson CJ (2009). Introducing mothur: open-source, platform-independent, community-supported software for describing and comparing microbial communities. Appl Environ Microbiol.

[33] Pruesse E, Quast C, Knittel K, Fuchs BM, Ludwig W, Peplies J, Glöckner FO (2007). SILVA: a comprehensive online resource for quality checked and aligned ribosomal RNA sequence data compatible with ARB. Nucleic Acids Res.

[34] Wang Q, Garrity GM, Tiedje JM, Cole JR (2007). Naive Bayesian classifier for rapid assignment of rRNA sequences into the new bacterial taxonomy. Appl Environ Microbiol.

[35] Caporaso JG, Kuczynski J, Stombaugh J, Bittinger K, Bushman FD, Costello EK, Fierer N, Peña AG, Goodrich JK, Gordon JI (2010). QIIME allows analysis of high-throughput community sequencing data. Nat Methods.

[36] Team, R. Core: R: A language and environment for statistical computing. R Foundation for Statistical Computing, Vienna, Austria. Online: http://www.R-project. org (2013):201. Accessed August 8^th^ 2016.

[37] Hajia M (2017). Limitations of different PCR protocols used in diagnostic laboratories: a short review. Mod Med Lab J.

[38] Wang A, Yao Z, Zheng W, Zhang H (2014). Bacterial communities in the gut and reproductive organs of *Bactrocera minax* (Diptera: Tephritidae) based on 454 pyrosequencing. PLoS One.

[39] Wang H, Jin L, Zhang H (2011). Comparison of the diversity of the bacterial communities in the intestinal tract of adult *Bactrocera dorsalis* from three different populations. J Appl Microbiol.

[40] Kuzina Lyudmila V., Peloquin John J., Vacek Don C., Miller Thomas A. (2001). Isolation and Identification of Bacteria Associated with Adult Laboratory Mexican Fruit Flies, Anastrepha ludens (Diptera: Tephritidae). Current Microbiology.

[41] Zhang Muqing, Powell Charles A., Benyon Lesley S., Zhou Hui, Duan Yongping (2013). Deciphering the Bacterial Microbiome of Citrus Plants in Response to ‘Candidatus Liberibacter asiaticus’-Infection and Antibiotic Treatments. PLoS ONE.

[42] Trivedi P, Spann T, Wang N (2011). Isolation and characterization of beneficial bacteria associated with citrus roots in Florida. Microb Ecol.

[43] Chandler JA, Lang JM, Bhatnagar S, Eisen JA, Kopp A (2011). Bacterial communities of diverse Drosophila species: ecological context of a host–microbe model system. PLoS Genet.

[44] Lauzon C, McCombs S, Potter S, Peabody N (2009). Establishment and vertical passage of *Enterobacter* (*Pantoea*) *agglomerans* and *Klebsiella pneumoniae* through all life stages of the Mediterranean fruit fly (Diptera: Tephritidae). Ann Entomol Soc Am.

[45] Behar A, Jurkevitch E, Yuval B (2008). Bringing back the fruit into fruit fly–bacteria interactions. Mol Ecol.

[46] Ley RE, Hamady M, Lozupone C, Turnbaugh PJ, Ramey RR, Bircher JS, Schlegel ML, Tucker TA, Schrenzel MD, Knight R (2008). Evolution of mammals and their gut microbes. Sci.

[47] Ochman H, Worobey M, Kuo C-H, Ndjango J-BN, Peeters M, Hahn BH, Hugenholtz P (2010). Evolutionary relationships of wild hominids recapitulated by gut microbial communities. PLoS Biol.

[48] Broderick NA, Raffa KF, Goodman RM, Handelsman J (2004). Census of the bacterial community of the gypsy moth larval midgut by using culturing and culture-independent methods. Appl Environ Microbiol.

[49] Hui X, Wei G-F, Jia S, Huang J, Miao X-X, Zhou Z, Zhao L-P, Huang Y-P (2006). Microbial communities in the larval midgut of laboratory and field populations of cotton bollworm (*Helicoverpa armigera*). Can J Microbiol.

[50] Yao Zhichao, Wang Ailin, Li Yushan, Cai Zhaohui, Lemaitre Bruno, Zhang Hongyu (2015). The dual oxidase gene BdDuox regulates the intestinal bacterial community homeostasis of Bactrocera dorsalis. The ISME Journal.

[51] Ben-Yosef M, Pasternak Z, Jurkevitch E, Yuval B (2015). Symbiotic bacteria enable olive fly larvae to overcome host defences. R Soc Open Sci.

[52] Thaochan N., Drew R. A. I., Hughes J. M., Vijaysegaran S., Chinajariyawong A. (2010). Alimentary Tract Bacteria Isolated and Identified with API-20E and Molecular Cloning Techniques from Australian Tropical Fruit Flies,Bactrocera cacuminataandB. tryoni. Journal of Insect Science.

[53] Robacker DC, Bartelt RJ (1997). Chemicals attractive to Mexican fruit fly from *Klebsiella pneumoniae* and *Citrobacter freundii* cultures sampled by solid-phase micro-extraction. J Chem Ecol.

[54] Lee CJ, Demilo AB, Moreno DS, Martinez AJ (1995). Analysis of the volatile components of a bacterial fermentation that is attractive to the mexican fruit-fly, *Anastrepha ludens*. J Agric Food Chem.

[55] Ben-Yosef M, Jurkevitch E, Yuval B (2008). Effect of bacteria on nutritional status and reproductive success of the Mediterranean fruit fly *Ceratitis capitata*. Physiol Entomol.

[56] Lloyd A, Drew R, Teakle D, Hayward A (1986). Bacteria associated with some Dacus species (Diptera: Tephritidae) and their host fruit in Queensland. Aust J Biol Sci.

[57] Liu L, Martinez-Sañudo I, Mazzon L, Prabhakar C, Girolami V, Deng Y, Dai Y, Li Z. Bacterial communities associated with invasive populations of *Bactrocera dorsalis* (Diptera: Tephritidae) in China. Bull Entomol Res. 2016;1. 10.1017/S0007485316000390 27600786

[58] Armougom F, Henry M, Vialettes B, Raccah D, Raoult D (2009). Monitoring bacterial community of human gut microbiota reveals an increase in Lactobacillus in obese patients and methanogens in anorexic patients. PLoS One.

[59] Taylor SM, He Y, Zhao B, Huang J (2009). Heterotrophic ammonium removal characteristics of an aerobic heterotrophic nitrifying-denitrifying bacterium, *Providencia rettger*i YL. J Environ Sci.

