# Supplementary figures and images for: Assessment of the Bacteria community structure across life stages of the Chinese Citrus Fly, Bactrocera minax (Diptera: Tephritidae)
Source: BMC Microbiol. 2019 Dec 24;19(Suppl 1):285. doi: 10.1186/s12866-019-1646-9 (PMC6929268; doi:10.1186/s12866-019-1646-9)

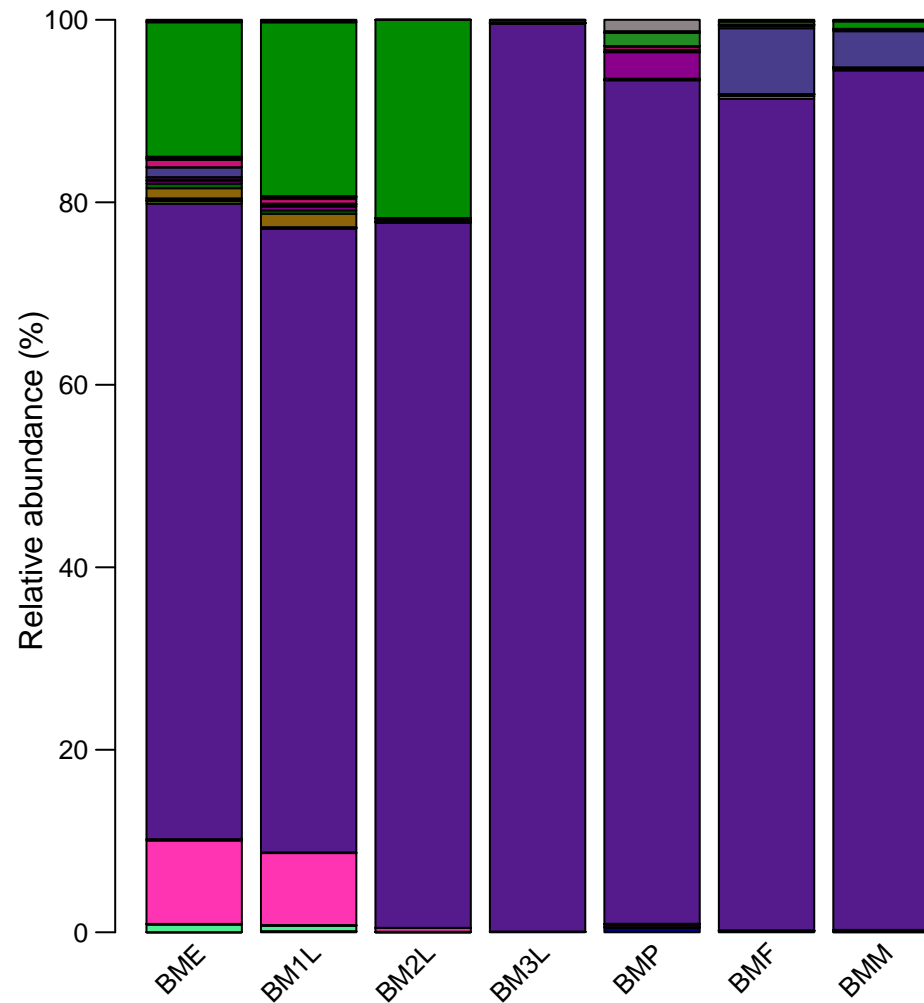

Supplement: Supplementary file 1 — Additional file 1. Relative abundance of taxa in the 16S rRNA libraries from different developmental stages of Bactrocera minax: BME- B. minax egg, BM1L- B. minax first instar larva, BM2L- B. minax second instar BM3L- B. minax third instar larva, BMP- B. minax pupa, BMF- B. minax female, BMM- B. minax male. Classification results were obtained from sequence alignment against RDP training set version 9 [34] and can be displayed for different taxonomic levels (Phylum; Class; Order; Family; Genus; Operational taxonomic units created at 97% sequence similarity) [file 12866_2019_1646_MOESM1_ESM.pdf]
